# Ursolic acid inhibits colistin efflux and curtails colistin resistant *Enterobacteriaceae*

**DOI:** 10.1186/s13568-019-0750-4

**Published:** 2019-02-18

**Authors:** Niranjana Sri Sundaramoorthy, Harihar M. Mohan, Shankar Subramaniam, Thiagarajan Raman, Subramaniapillai Selva Ganesan, Aravind Sivasubamanian, Saisubramanian Nagarajan

**Affiliations:** 1 Center for Research on Infectious Diseases, SCBT, SASTRA Deemed University, Thanjavur, Tamil Nadu India; 2 Department of Microbiology and Immunology, University of Michigan Medical School, Ann Arbor, MI 48109 USA; 3 School of Chemical and Biotechnology, SASTRA Deemed University, Thanjavur, Tamil Nadu India; 4 Research Center for Food Safety and Nutrition, School of Agriculture and Biology, Shanghai Jiao Tong University, Shanghai, China; 5 Advanced Zoology and Biotechnology Department, Ramakrishna Mission Vivekananda College, Chennai, Tamil Nadu India

**Keywords:** Synergy, Ursolic acid, Colistin resistance, *Klebsiella pneumoniae*, *E. coli*, Zebrafish infection

## Abstract

Colistin resistance in *Enterobacteriaceae* especially *Klebsiella pneumoniae* and *Escherichia coli* is driving the evolution of pan drug resistant strains. Screening a library of 13 plant nutraceuticals led to the identification of acetyl shikonin and ursolic acid, which exhibited synergy with colistin against extremely drug resistant (XDR) clinical strains of *E. coli* (U3790) and *K. pneumoniae* (BC936). Ursolic acid caused a significant colistin MIC reversal of 16-fold in U3790 and 4-fold in BC936 strains. Ursolic acid also potentiated the bactericidal effect of colistin against both U3790 and BC936 by causing ~ 4 to 4.5 log fold decline in CFU of both clinical isolates in a time kill assay. At 2× minimum effective concentration, ursolic acid was non-toxic to zebrafish as evidenced by brain and liver enzyme profiles and by histopathology studies. In combination with colistin, ursolic acid reduced bacterial bioburden of U3790/BC936 by 1–1.58 log fold from the infected muscle tissue of zebrafish. Mechanistic explorations via studies on real time efflux, membrane potential and intracellular accumulation of dansyl chloride tagged colistin revealed that colistin efflux is inhibited by ursolic acid. In addition, ursolic acid also enhanced outer membrane permeability which probably facilitates colistin’s attack on outer and inner membranes. Our study shows that ursolic acid synergizes with colistin by inhibiting colistin efflux in *Enterobacteriaceae* that helps to curtail colistin resistant *Enterobacteriaceae*.

**Electronic supplementary material:**

The online version of this article (10.1186/s13568-019-0750-4) contains supplementary material, which is available to authorized users.

## Introduction

Multi drug resistant *Enterobacteriaceae* especially *Klebsiella pneumoniae* and *Escherichia coli,* are the leading causes of mortality and morbidity in neonatal bacterial sepsis caused by Gram negatives. Roughly 54% of *K. pneumoniae* and 38% of *E. coli* strains that caused neonatal sepsis were observed to be multi drug resistant (Investigators of the Delhi Neonatal Infection Study (DeNIS) collaboration 2016). Colistin is regarded as a drug of last resort in therapeutic management of Gram negative infections (Yau et al. 2009) and colistin resistance in carbapenem resistant *Enterobacteriaceae* implies that we are in fact dealing with pan drug resistant strains, with very limited/no therapeutic options. Colistin resistance was known to be chromosomally mediated (Yau et al. 2009). But of late, studies have shown that plasmid encoded *mcr*-*1* gene harbored by *E. coli* SHP47 confers colistin resistance in farm animals in China (Liu et al. 2016), subsequently other reports have also highlighted spread of plasmid mediated colistin resistance in Europe (Skov and Monnet 2016). Resistance to colistin is typically caused by modification of LPS with 4-amino 4-dexoy arabinose or with phosphoethanolamine both of which alters surface charge, ultimately resulting in reduced binding of colistin to outer membrane of the bacteria (Olaitan et al. 2014). Among *Enterobacteriaceae,* especially with *Klebsiella pneumoniae* clinical isolates, mutation/disruption of *mgrB* was reported as the most common reason for colistin resistance (Cannatelli et al. 2014).

Due to resistance to last resort drugs like colistin, infections by MDR *Enterobacteriaceae* are associated with treatment failure and high mortality. Hence, restoring colistin sensitivity is likely to improve therapeutic outcomes significantly. Towards this end, we were interested to explore ability of natural products to interact synergistically with colistin and augment bactericidal effect of colistin in clinical isolates of *Enterobacteriaceae* (especially *E. coli* and *Klebsiella pneumoniae*.) both in vitro and in vivo in a zebrafish infection model that we and others have developed (Christena et al. 2016; Cheepurupalli et al. 2017). In addition, we were also interested in exploring mechanism of action of plant metabolites that displays synergistic interaction with colistin.

## Materials and methods

### Strains and compounds

*Klebsiella pneumoniae* reference strain (MTCC 432: *K. pneumoniae*-1) was procured from Microbial Type Culture Collection (MTCC) Chandigarh, India and *E. coli* MG1655 was a kind gift from Dr. Aswin Sai Narayan Seshasayee NCBS, Bangalore. The clinical isolates *of Klebsiella pneumoniae* and *Escherichia coli* were obtained from Sundaram Medical Foundation (SMF), Chennai, India. The *K. pneumoniae* isolates are designated as (BC936, E474, BC1415, U2016, BC1994, BC2412, U3866) and the *E. coli* isolates as (U3176 and U3790). All the antibiotics, media and chemicals employed in the study were purchased from Sigma Aldrich, USA, Alfa-Aesar, USA or HiMedia, India. The plant metabolites used as test compounds was from a natural product library which include caffeic acid, naringin, naringenin, arjunolic acid, ursolic acid, acetyl shikonin, β-methylacrylshikonin, chrysin, chrysophenol, ventilone, ventiloquinone, emodin and physcion. Stocks for the test compounds were freshly prepared in DMSO and stored at − 20 °C for further use.

### Antimicrobial studies

All the test compounds were screened for their minimum inhibitory concentration (MIC) by microbroth 2-fold-dilution method to check for the antimicrobial efficacy against the *E. coli* and *K. pneumoniae* strains as reported earlier (Andrews and Andrews 2001). Similarly, the susceptibility pattern of other clinical isolates towards other antibiotics was also analyzed.

### Synergy and modulation of antibiotic resistance

To understand the combinatorial activity, plant metabolites and antibiotics were used in combination at different concentrations by checkerboard assay against reference and clinical isolates as reported earlier (Lowrence et al. 2016). The Fractional inhibitory concentration (FIC) index was calculated and if FIC values are < 0.5, the interaction is synergistic, 0.5–2.0, interaction is additive and > 2, the interaction is antagonistic (Odds 2003). The colistin potentiating ability of ursolic acid against colistin resistant isolates of *E. coli* and *K. pneumoniae* was evaluated. Ursolic acid at sub-MIC concentration was used along with varying concentrations of colistin and incubated for 18–24 h at 37 °C. The fold reduction in MIC (MIC reversal) of colistin when combined with ursolic acid was determined as modulation factor as reported earlier (Lowrence et al. 2016; Sundaramoorthy et al. 2018).

### Real time efflux study

To analyze the efflux pump inhibitory activity of ursolic acid against *Klebsiella pneumoniae* and *Escherichia coli*, real time efflux studies were performed using ethidium bromide as a substrate. The cells were de-energized, EtBr was added and then glucose was added to re-energize the cells, which would activate efflux. Resulting fluorescent intensity was measured with Ex 360 nm and Em 590 nm as reported earlier (Sundaramoorthy et al. 2018). The increase in fluorescent intensity of EtBr was taken as a measure of efflux inhibition activity.

### Time kill assay

Bactericidal effect of ursolic acid in combination with colistin was evaluated against the XDR clinical isolates U3790 and BC936 by time kill assay (Belley et al. 2008; Grillon et al. 2016). Early log phase cells were subjected to following treatments, viz., colistin (4 μg/ml) and colistin (4 μg/ml) + ursolic acid (40 µM). Untreated culture was maintained as a growth control. The samples were withdrawn at different time intervals (0, 1, 2, 3, 4, 5 and 24 h), serially diluted and plated on agar plates. The plates were incubated at 37 °C for 24 h and from plate counts, Colony forming units (CFU)/ml was calculated and bactericidal effect of combination was discerned.

### Membrane permeability and integrity assay

#### NPN-assay

The ability of ursolic acid to permeabilize outer membrane of *Enterobacteriaceae* was assessed by 1-*N*-phenylethylamine (NPN) uptake assay as reported earlier (Helander and Mattila-Sandholm 2000). NPN exhibits enhanced fluorescence in phospholipid environment. Since the outer membrane (OM) of Gram negative bacteria affords steric hindrance to hydrophobic molecules and prevents NPN entry due to LPS, increased NPN fluorescence due to treatment, indicates enhanced OM permeability. Briefly, cells were grown to mid-log phase collected and washed with 5 mM HEPES buffer containing 0.2% glucose at pH 7.5 and resuspended in an equal volume of the same buffer. NPN was added at a concentration of 0.5 mM, this was immediately followed by addition of ursolic acid. Fluorescence due to NPN was measured (Ex 350 and Em 420 nm) using spectrofluorimeter (JASCO FP-8500, Jasco, Tokyo, Japan). NPN in buffer and NPN in buffer along with cells were maintained as controls.

#### Membrane integrity assay

Compromise in cell membrane integrity due to treatment with ursolic acid was assessed as reported previously (Marks et al. 2013). Briefly, cells after treatment were collected at different time points (0, 1, 2, 3 and 4 h), pelleted at 13,250 rcf for 5 min. The release of DNA and proteins, due to loss of inner membrane integrity, was quantified by measuring absorbance at 260 nm and 280 nm respectively using UV–Vis Spectrophotometer (Evolution 201, Thermoscientific, USA). Treatment with 0.5% Triton X 100 was used as a positive control.

### Membrane potential assay

The effect of ursolic acid alone/with colistin in perturbing membrane potential was evaluated using DiSc3, a cationic membrane permeabilizing dye. Intact bacterial cells accumulate the dye in the lipid bilayer, resulting in quenching of fluorescence. When the membrane gets depolarized, dye gets released to the surrounding aqueous phase and fluorescence gets enhanced (Te Winkel et al. 2016). The fluorescent intensity (Ex 610 ± 5 nm and Em 660 ± 5 nm) of buffer with DiSc3 (1 μM) was measured initially. Mid log cells were added, which reduces the fluorescent intensity due to accumulation of dye in cells. Colistin, ursolic acid and colistin with ursolic acid treatments were given and the resulting variation in fluorescence intensity due to various treatments was quantified using spectrofluorimeter (JASCO FP-8500, Jasco, Tokyo, Japan).

### ROS assay

Release of reactive oxygen species (ROS) from XDR *E. coli* and *K. pneumoniae* clinical isolates, in the presence of colistin and ursolic alone and in combination was discerned using fluorophore Dichloro-dihydro-fluorescein diacetate (DCFH-DA) and fluorescence of ROS induced dichlorofluorescein (DCF) formation was quantified using a fluorescence spectrophotometer (JASCO FP-8500, JASCO, Tokyo, Japan) (Ex 485 nm and Em 538 nm).

### Colistin accumulation studies

In order to visualize the intracellular accumulation of colistin within the cells, colistin was conjugated using the fluorophore dansyl chloride as reported earlier (Soon et al. 2011). Intracellular accumulation of dansyl chloride conjugated colistin in response to different treatments viz., colistin-dansyl chloride, colistin-dansyl chloride + ursolic acid, colitin-dansyl chloride + CCCP was evaluated using fluorescent microscopic imaging (Nikon eclipse Ni-U, Nikon, Tokyo, Japan), to discern effect of various treatments on colistin accumulation.

### Fish toxicity studies

All experiments were performed in compliance with applicable national and/or institutional guidelines for the care and use of animals (Animal Biosafety Level 2). Adult zebrafish (*Danio rerio*), either male/female, measuring 4 to 5 cm in length, weighing approx. 300 mg, were purchased from a local aquarium in Thanjavur, India. Animal acclimatization was performed as reported earlier (Westerfield 1995). To evaluate the effect of ursolic acid on brain and liver enzyme profiles of zebrafish, a total of 10 fish were exposed to 32 mg/L of the respective compounds for 48 h. At the end of exposure (48 h), fish were sacrificed (anesthetized by 150 mM MS-222 and euthanized by decapitation), skin removed and the liver/brain from two fish from the same group were pooled and homogenized in ice-cold buffer (Tris–HCl, 0.1 M, pH 7.4). The homogenate was centrifuged (10,000×*g*, 10 min, 4 °C) and supernatant used for all analyses in duplicates. Protein was estimated by the method of Lowry et al. (1951). Estimation of carboxyl esterase was essentially as described by Argentine and James (Argentine and James 1995) and acetylcholinesterase (AChE) activity was measured by Edmann’s degradation. Histopathology of ursolic acid injected fish was performed to analyze any histopathological alterations. The fish were sacrificed and fixed with 10% formalin. Thin sections were made after embedding process, stained with hematoxylin–eosin and viewed and imaged using a bright field microscope (Nikon Eclipse Ni-U, Japan).

### Zebrafish infection

Intramuscular infection of zebrafish (n = 6) with colistin resistant *Klebsiella pneumoniae* BC936 and *Escherichia coli* U3790 strains corresponding to OD of 0.2 (~ 1 × 10^6^ CFU/ml) was performed as reported earlier (Neely et al. 2002) with slight modifications. 2 h post infection, compounds viz., ursolic acid/colistin alone and ursolic acid +colistin combination were administered via intramuscular injection as a single dose. 48 h post treatment, fish were euthanized, decapitated, muscle tissue was dissected, minced, serial diluted and plated onto LB agar to discern colony counts after 24 h of incubation. Based on cell counts, graph was plotted and ability of ursolic acid alone and in combination with colistin to reduce bacterial bioburden in infected muscle tissue was estimated.

## Results

### Antimicrobial profiling of isolates

The clinical isolates of *Klebsiella pneumoniae* and *Escherichia coli* were obtained from blood of unrelated patients diagnosed with bacterial sepsis from the same health care setting albeit at different time points. Antimicrobial profiling of clinical isolates showed that *K. pneumoniae* BC2412, U3866 and *E. coli* U3176 strains were relatively more sensitive to colistin whereas *K. pneumoniae* BC936 and *E. coli* U3790 were the most colistin resistant strains, which also exhibits a very high MIC to most of the other antibiotics evaluated (Additional file 1: Table S1). Based on its resistance profile, both *E. coli* U3790 and *K. pneumoniae* BC936 strains were deemed as extremely drug resistant (XDR) strains as proposed by international expert committee for standard definitions on acquired resistance (Magiorakos et al. 2012). We observed that U3790 and BC936 had a high colistin MIC (32 µg/ml), rest of the strains displayed an MIC (1–8 µg/ml) for colistin. MIC of plant metabolites was determined against the XDR U3790 and BC936 strains wherein, most plant metabolites tested exhibited a higher MIC ranging from 256 to 1028 µg/ml (Additional file 1: Table S2).

### Synergy testing

Synergy between the plant metabolites and colistin was evaluated by checkerboard assay against U3790 and BC936. Among the metabolites evaluated, only acetyl shikonin and ursolic acid displayed synergy with U3790 and BC936 strains, the rest of the metabolites exhibited additive effect and emodin exhibited antagonistic effect. Both acetyl shikonin and ursolic acid were further evaluated for their ability to synergize with colistin against all strains employed in this study. Among the two metabolites chosen, ursolic acid at 32 µg/ml displayed synergy with colistin for most of the strains tested, other than one strain *K. pneumoniae* (BC1415) wherein FIC index values were  < 0.5. But acetyl shikonin displayed synergy only with four strains and for rest five strains, it exhibited an additive effect and hence it was not taken up further (Table 1).

**Table 1:** Ursolic acid exhibits synergy with colistin against most of the strains of *Escherichia coli* and *Klebsiella pneumoniae*

	Col + UR		Col + AS	
	FIC index	Effect	FIC index	Effect
*E. coli*				
U3790	0.19	*Synergy*	0.07	*Synergy*
U3176	0.19	*Synergy*	0.37	*Synergy*
*K. pneumoniae*				
BC936	0.06	*Synergy*	0.06	*Synergy*
U2016	0.50	*Synergy*	0.25	*Synergy*
BC1415	1.06	Additive	1.03	Additive
BC1994	0.38	*Synergy*	0.62	Additive
MTCC *K. pneumoniae*	0.38	*Synergy*	1.25	Additive
BC2412	0.38	*Synergy*	1.25	Additive
U3866	0.25	*Synergy*	1	Additive

*Synergy* implies FIC values < 0.5

### Reversal of colistin MIC using ursolic acid

Ability of ursolic acid to reduce colistin MIC in all clinical isolates was evaluated. Except for BC1415 strain of *K. pneumoniae*, with which ursolic acid did not display synergy and hence, no decrease in colistin MIC was observed, for rest of the 8 strains (both *E. coli* and *K. pneumoniae*), ursolic acid caused 2–16-fold reduction in colistin MIC (Table 2). A 4-fold reduction in colistin MIC was seen for *K. pneumoniae* strains U3866, MTCC reference strains and BC936 strain whereas, 16-fold reversal in colistin MIC was observed for the *E. coli* U3790 strain. Thus by virtue of its synergistic interaction, ursolic acid was able to potentiate anti-bacterial effect of colistin in various clinical isolates of *Escherichia coli* and *K. pneumoniae.*

**Table 2:** Ursolic acid potentiates bacteriostatic effect of colistin in clinical isolates of *Escherichia coli* and *Klebsiella pneumoniae*

Strains	MIC (μg/ml)		
	Col	Col + UR	Modulation factor
*E. coli*			
U3790	32	2	16
U3176	1	0.5	2
*K. pneumoniae*			
BC936	32	8	4
U2016	8	4	2
BC1415	8	8	1
BC1994	2	1	2
MTCC *K. pneumoniae*	2	0.5	4
BC2412	1	0.5	2
U3866	1	0.125	4

### Time kill curve

**Fig. 1:**
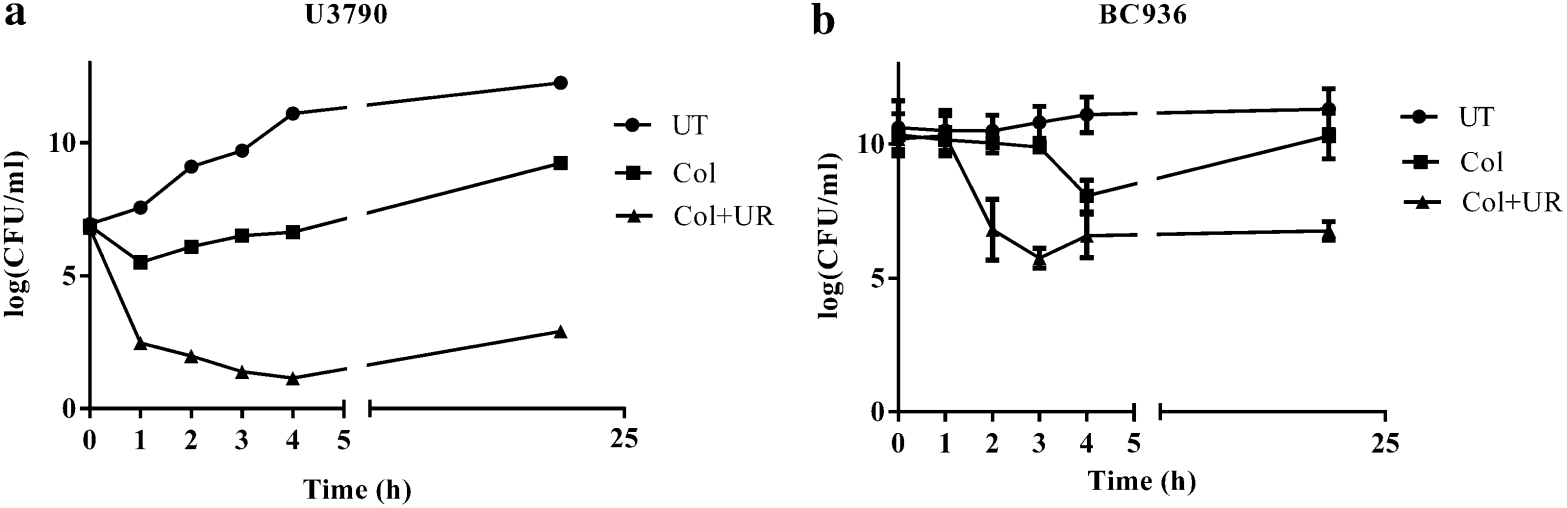
Time kill curve: UR potentiates the effect of colistin and causes growth reversal in resistant isolates of **a** *E. coli* U3790, **b** *K. pneumoniae* BC936. The culture was treated with colistin (Col—filled square) alone and colistin with ursolic acid (filled triangle). The samples were withdrawn at time intervals from 0, 1, 2, 3, 4 and 24 h and plated on to LB agar plates. The error bar represents the standard error of mean of three independent samples

In order to validate ability of ursolic acid to potentiate the bactericidal effect of colistin, a time kill assay was performed. Overnight grown culture of U3790 and BC936 were diluted to 0.1 OD and was subjected to following treatments: colistin alone, colistin and ursolic acid (16 µg/ml) and untreated sample was maintained as a growth control. In *E. coli* U3790 strain, 24 h plate counts revealed a 5–6 log increase in cell counts in the untreated control relative to starting population (Fig. 1a). Treatment with colistin caused an initial decline by 1 h, following which re-growth was observed, and by 24 h ~ 2 log CFU increase in cell count was observed relative to initial population. When ursolic acid was administered along with colistin, a steep decline in cell counts was noted by 1 h and by 24 h, a decline in cell count of ~ 4 log CFU relative to founder population was maintained (Fig. 1a). Similarly for *K. pneumoniae* BC936 strain, colistin treatment caused an initial decline at 3 h, but regrowth was observed by 24 h (Fig. 1b). Ursolic acid in combination with colistin caused a ~ 4.5-fold reduction in bioburden, which was maintained until 24 h, implying that ursolic acid effectively potentiated bactericidal effect of colistin and restricted growth of both U3790 and BC936 strains for 24 h.

### Zebrafish toxicity testing

**Fig. 2:**
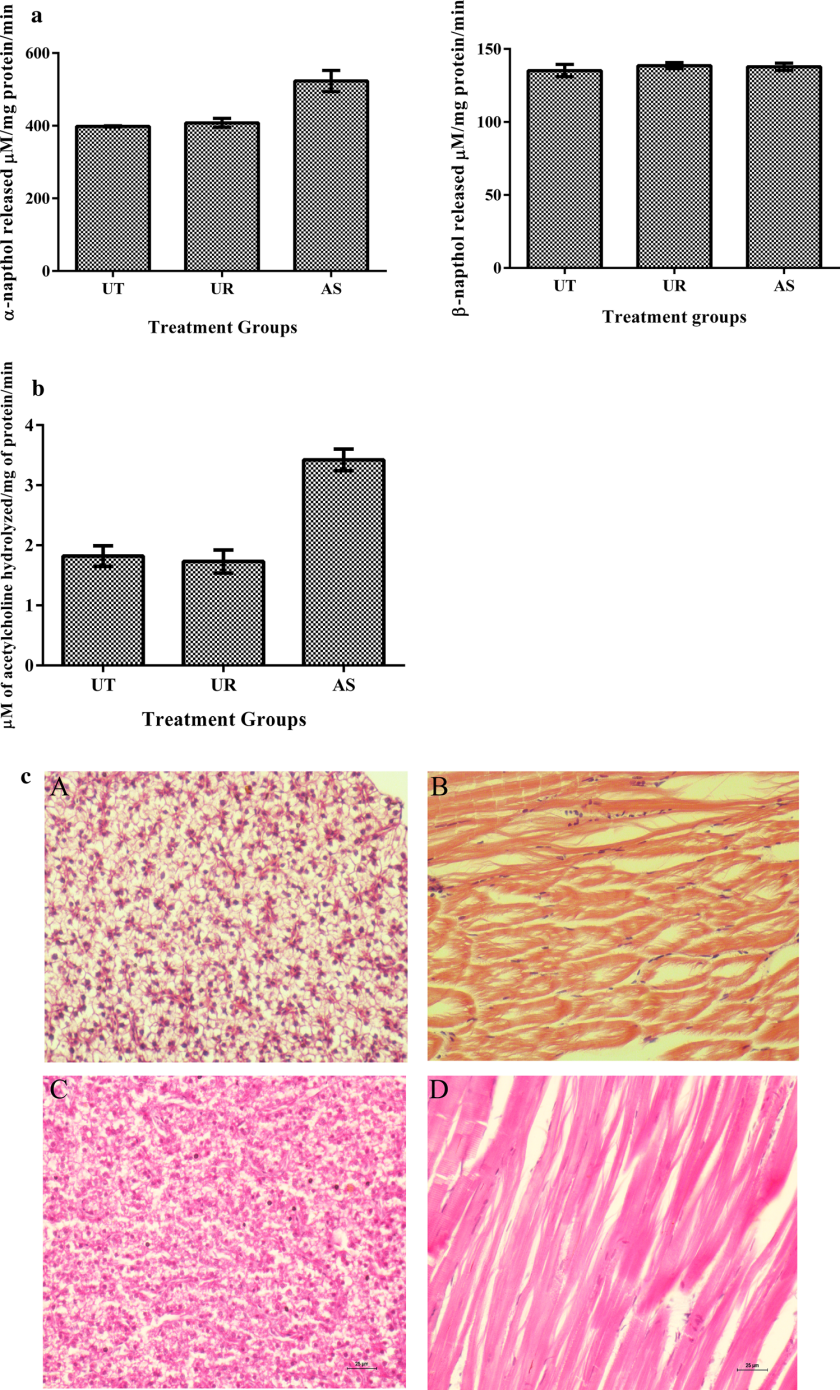
**In vivo toxicity testing:** Impact of ursolic acid on liver (**a**) and brain (**b**) enzyme activities. Liver α carboxyl esterase  and brain acetyl choline esterase activities were carried out using pooled liver/brain tissues from different fishes in the same group after 48 h of treatment. **c** Histopathology analysis: fish were injected with PBS and ursolic acid intramuscularly. After 48 h, the fish were sacrificed and preserved immediately in 10% formalin. They were embedded in paraffin blocks, sectioned, stained with Hematoxylin and eosin and imaged using bright field microscope. A–D represents histopathology of liver and muscle of untreated and ursolic acid (UR) treated respectively

Toxicity of ursolic acid was evaluated using by determining liver and brain enzyme profiles of zebrafish, because relative to in vitro cell culture based toxicity evaluation, whole organism based toxicity testing is likely to yield holistic insights (Astashkina et al. 2012). Liver and brain enzyme levels due to ursolic acid treatment were comparable as that of the untreated control implying that ursolic acid was non-toxic at the concentration tested (Fig. 2a, b). Whereas acetyl shikonin treatment resulted in enhanced acetylcholine esterase activity and alpha-naphthol levels indicating toxicity. Histopathological analysis of liver and muscle from ursolic acid injected fish tissue sections revealed no signs of inflammation, neutrophil accumulation or tissue damage and tissue appeared similar to untreated control, showing non-toxic nature of ursolic acid at the tested concentration (Fig. 2c).

### Zebrafish infection study

**Fig. 3:**
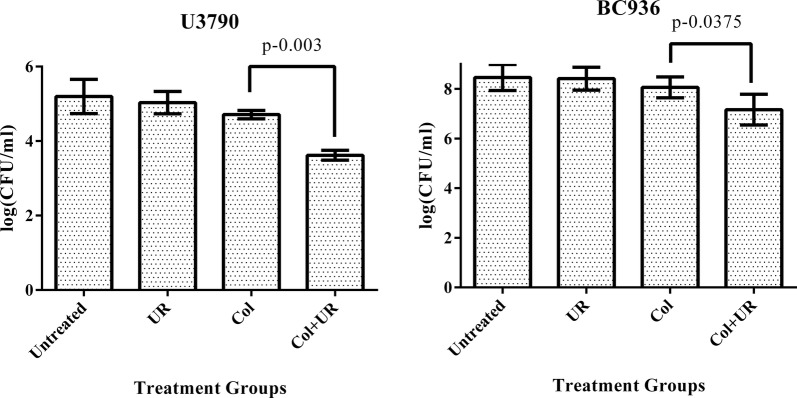
Ursolic acid synergistically potentiates bactericidal effect of colistin in infected zebrafish. *E. coli* U3790 and *K. pneumoniae* BC936 were intramuscularly injected in zebrafish and treated with either colistin (Col)/ursolic acid (UR) or colistin + ursolic acid in combination for 24 h. Following treatment, fish were euthanized and the muscle tissue was collected in PBS, homogenized, serially diluted and plated onto LB agar. The colony counts was scored after 24 h and represented as log CFU/ml. The error bar represents the standard error of mean of three independent samples

Zebrafish (n = 6 per group) was infected independently with ~ 1 × 10^6^ CFU/ml (corresponding to an OD of 0.2) of  *E. coli* U3790 and *K. pneumoniae* BC936 strains and 2 h post infection, fish were subjected to various treatments viz., colistin alone, ursolic acid alone and colistin and ursolic acid. Plate counts from infected muscle tissue of various groups showed that untreated control displayed a colony count of 5.2 log CFU. Colony counts in case of ursolic acid treatment group was comparable to untreated control at 5.7 log CFU. Colistin treatment resulted in a decline in plate count to 4.7 log CFU. When ursolic acid was administered in combination with colistin, a further reduction in plate count to 3.62 log CFU was observed for U3790 strain. With BC936 strain, treatment with colistin did not cause significant decline in cell counts relative to infected and untreated cells. The combination of ursolic acid and colistin caused 1 log decline in plate counts. Most importantly treatment with combination caused statistically significant decline in cell counts for both *E. coli* U3790 (P = 0.003) and *K. pneumoniae*- BC936 (P = 0.0375), although the reduction in cell counts was more pronounced for U3790 strain. Thus relative to untreated control, ursolic acid-colistin combination caused 1.1 to 1.58 log CFU reduction in both U3790 and BC936 strains (Fig. 3). Reduction in bioburden following combinatorial treatment highlights the ability of ursolic acid in potentiating bactericidal effect of colistin in vivo.

### Membrane permeability

NPN was used as fluorophore to evaluate membrane permeability. In *E. coli* U3790, colistin treatment caused a slight increase in membrane permeability relative to untreated control and ursolic acid caused ~ 1.5-fold increase in membrane permeability. But when ursolic acid was mixed with colistin a significant 2.5-fold increase in membrane permeability was observed (Additional file 1: Fig S1). With *K. pneumoniae* BC936 strain, colistin treatment showed ~ 1-fold increase in membrane permeability whereas ursolic acid caused ~ 0.8-fold increase in permeability. Combination of colistin with ursolic acid resulted in a 2.4-fold increase in membrane permeability. Thus ursolic acid synergistically enhanced membrane permeabilizing effect of colistin in both U3790 and BC936 strains. By enhancing membrane permeability ursolic acid would afford more access to colistin and is likely to potentiate bactericidal effect of colistin.

### Real time efflux studies (RTE)

**Fig. 4:**
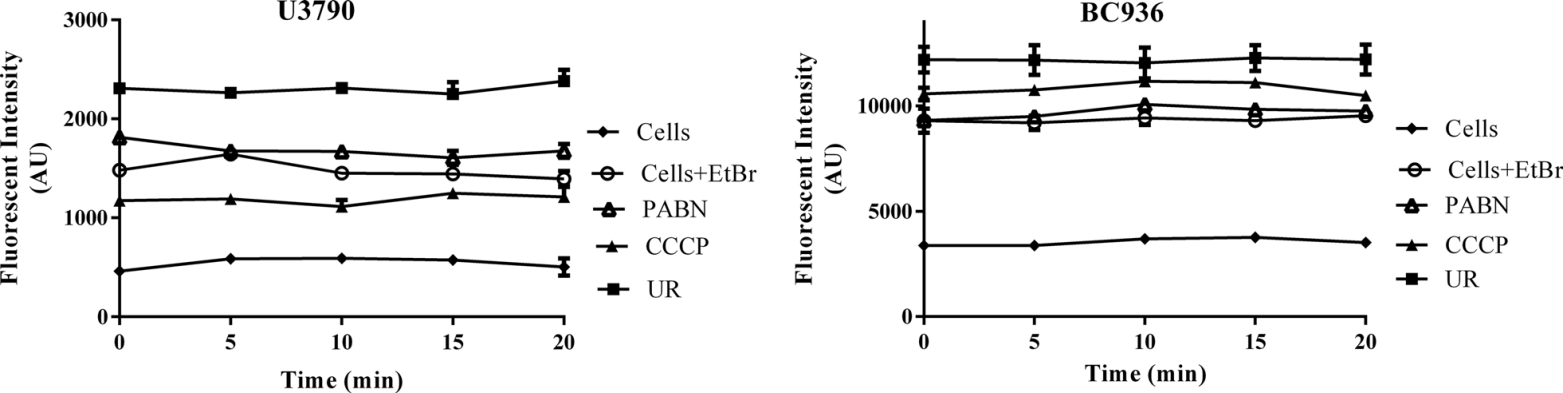
Ursolic acid inhibits efflux pump in colistin resistant clinical isolates *K. pneumoniae* BC936 and *E. coli* U3790. The deenergized cells of *K. pneumoniae* BC936 and *E. coli* U3790 were treated with ursolic acid (UR) for 1 h. The cells were then reenergized with glucose and residual fluorescence of EtBr was measured for a time period of 0–20 min. Phenyl arginine beta naphthylamide (PABN) and Carbonyl cyanide m-chlorophenylhydrazine (CCCP) were maintained as positive controls (Filled diamonds—untreated cells; Empty circles—cells + EtBr; Empty triangles—PABN treated; Filled triangles—CCCP treated; Filled squares—ursolic acid treated). The experiment was performed in triplicates

To discern the ability of ursolic acid to inhibit efflux pumps, real time efflux assay was performed for U3790 and BC936 strains. De-energized cells were incubated with ursolic acid for 1 h and then re-energized with glucose and fluorescence of EtBr was measured over a time course of 20 min. PAßN and CCCP were maintained as positive controls. RTE results revealed that ursolic acid caused enhanced inhibition of EtBr efflux relative to positive controls PAßN and CCCP (Fig. 4). Thus ursolic acid apart from enhancing membrane permeability, also inhibits efflux pumps in *Enterobacteriaceae.*

### Measurement of membrane potential

**Fig. 5:**
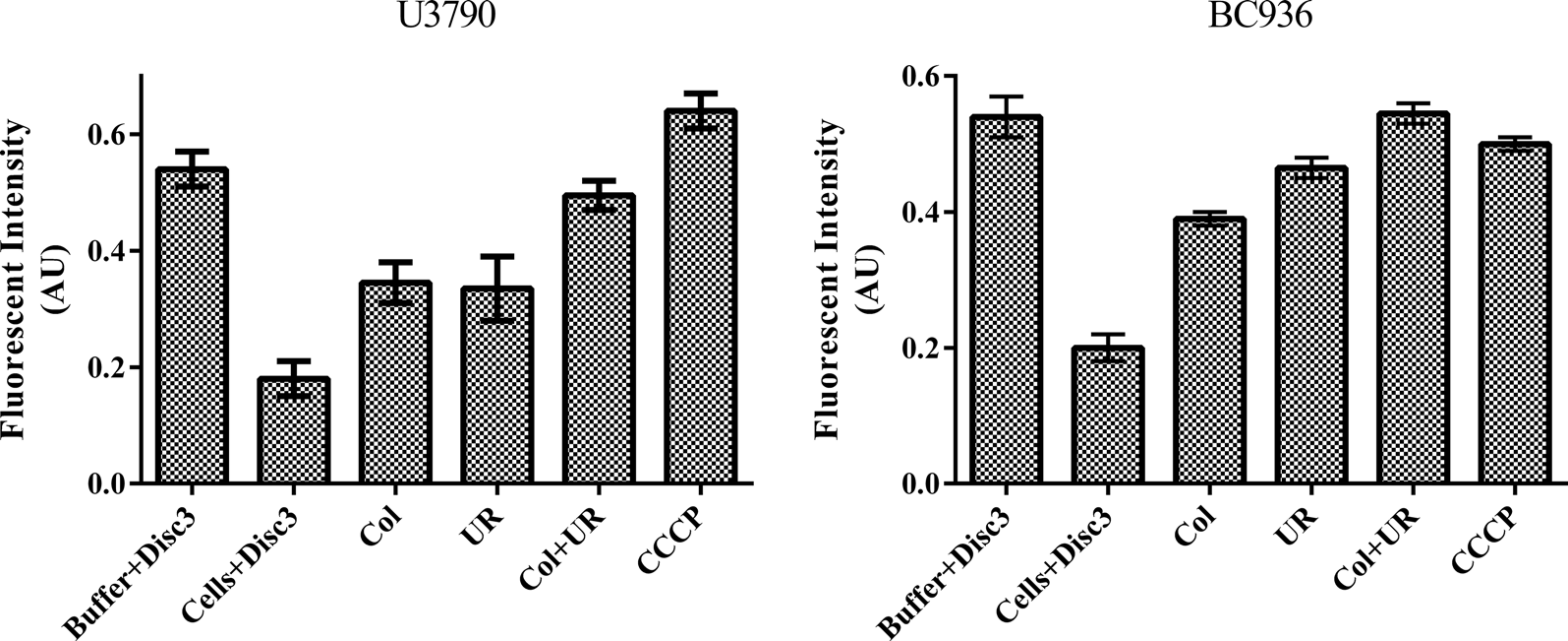
Ursolic acid with colistin causes effective membrane depolarization in *E. coli* U3790 and *K. pneumoniae* BC936. Mid log cells of U3790 and BC936 were treated with colistin (Col), ursolic acid (UR) and colistin + ursolic acid (Col + UR) in the presence of DiSc3. The fluorescent intensity was measured at Ex 605 nm and Em 665 nm

DiSc3 was used to determine the effect of ursolic acid with colistin on membrane potential. DiSc3 exhibits enhanced fluorescence in aqueous milieu and its fluorescence gets quenched when it partitions to lipid membranes of cells, compounds that alters membrane potential causes DiSc3 to partition back into aqueous environment resulting in enhanced fluorescence. In *E. coli* U3790 strain, addition of colistin and ursolic acid alone caused depolarization resulting in enhanced fluorescence. Combination of colistin with ursolic acid showed 2.5-fold enhanced depolarization effect relative to the individual components (Fig. 5). Even in *K. pneumoniae* BC936 strain, ursolic acid itself caused efficient depolarization, which was comparable to that of positive control CCCP. Colistin with ursolic acid showed ~ 2-fold increase in depolarization effect. This reveals that ursolic acid with colistin can disrupt membrane potential in both U3790 and in BC936 strains (Fig. 5). Membrane potential perturbation will abolish proton motive force, which energizes most of efflux transport proteins and hence by perturbing membrane potential ursolic acid inhibits efflux in *Enterobacteriaceae*.

### Colistin accumulation studies

**Fig. 6:**
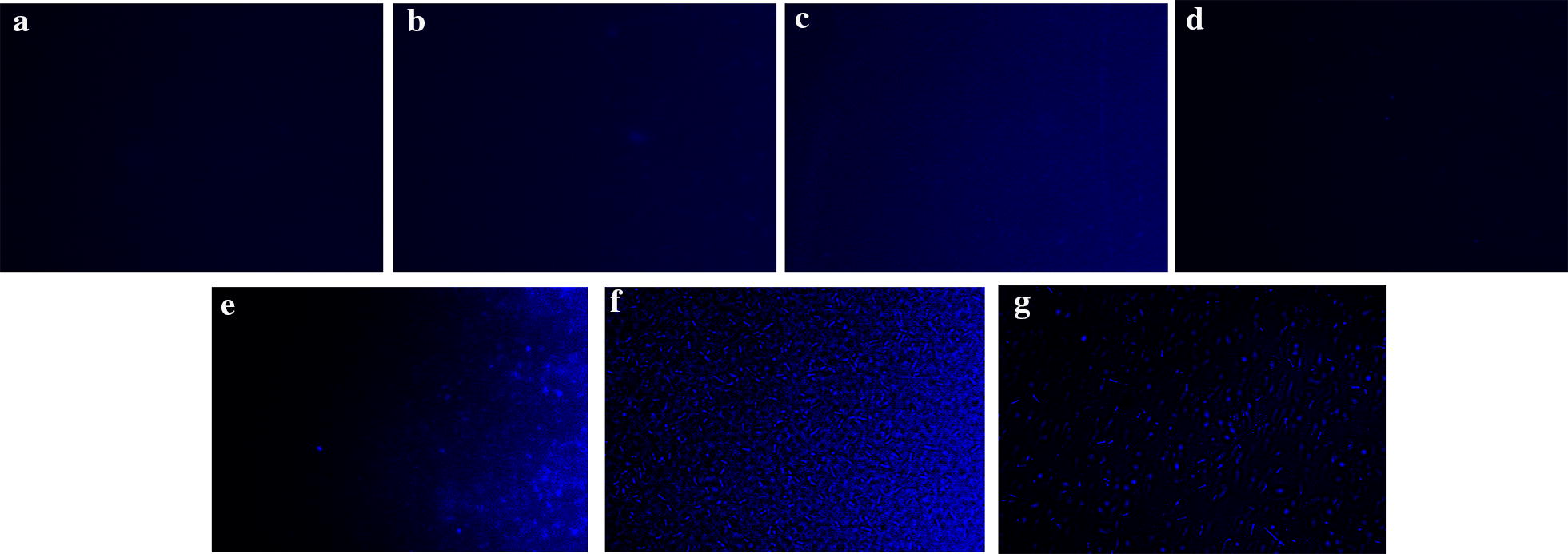
Colistin accumulation is enhanced in the presence of ursolic acid similar to CCCP. Mid log cells were incubated with colistin-dansyl chloride alone and along with ursolic acid (UR)/CCCP for 3 h and imaged using a fluorescent microscope. **a** Untreated, **b** colistin, **c** colistin + UR, **d** dansyl chloride, **e** colistin-dansyl chloride, **f** colistin-dansyl chloride + UR, **g** colistin-dansylchloride + CCCP

In order to confirm whether ursolic acid prevents colistin efflux, we qualitatively determined intracellular accumulation of colistin by tagging colistin with dansyl chloride, which exhibits fluorescence and can be imaged using a fluorescent microscope. Incubation of colistin-dansyl chloride with bacterial cells in the presence and absence of ursolic acid for 3 h showed that there is enhanced uptake of colistin-dansyl chloride in the presence of ursolic acid (Fig. 6), which was comparable to accumulation of colistin-dansyl chloride seen in the presence of positive control (CCCP). This shows that colistin indeed gets accumulated within the cells in presence of ursolic acid, which could be attributed to efflux inhibitory potential of ursolic acid primarily mediated by its ability to disrupt proton motive force.

### ROS generation

DCFH-DA was used as fluorophore to evaluate ROS production and in the presence of ROS, non-fluorescent DHFCA would be converted to strongly fluorescent DHF. Ursolic acid treatment caused only a minimum or negligible level of ROS production in bacterial cells relative to untreated cells. When treated with colistin, ~ 3–4-fold increases in ROS production was observed. Whereas when ursolic acid was combined with colistin there was reduction in ROS generation by ~ 1-fold in both U3790 and BC936 strains (Additional file 1: Fig S2). Thus ursolic acid treatment quenches ROS production in *Enterobacteriaceae.* Overall ursolic acid enhances bactericidal effect of colistin by enhancing outer membrane permeability and by disrupting proton motive, which prevents colistin efflux and results in increased intracellular accumulation of colistin, thereby leading to enhancement in bactericidal effect of colistin both in vitro and in vivo.

## Discussion

The recent declaration of list of drug resistant priority pathogens by WHO urging R&D to develop new antimicrobials, highlights the urgent need for addressing the issue of antimicrobial resistance. Incidentally as per World Health Organization (WHO) list, carbapenem resistant *Enterobacteriaceae* viz*., Klebsiella pneumoniae and Escherichia coli* belongs to the high priority category, which requires urgent attention by researchers to develop new antimicrobial agents or resistance modulatory agents. Pentacyclic triterpenoids such as oleanolic acid and ursolic acid and their derivatives were discovered earlier as potential alternatives to antibiotics against a broad spectrum of pathogens (Wolska et al. 2010; Wang et al. 2016). Ursolic acid was shown to display anti-*Mycobacterium tuberculosis* activity and immune-stimulatory activity by decreasing the microbial load in TB induced mice. Ursolic acid derivatives were also reported earlier to inhibit multidrug resistant *E. coli* when used in combination with kanamycin (do Nascimento et al. 2014) and were also shown to inhibit acrAB mediated efflux of nalidixic acid in *E. coli.* Ursolic and oleanic acids were earlier reported to synergize with beta lactam antibiotics against gram positive pathogens *S. aureus, S. epidermidis* and *Listeria monocytogenes* (Kurek et al. 2012). In a recent interesting study, ursolic and oleanic acids present in dichloromethane extract of shea butter tree were shown to synergize with ampicillin and oxacillin against MRSA and cause reversion of MRSA phenotype which was attributed to delocalization of PBP2 from the septal division site leading to disturbance in peptidoglycan synthesis, in addition the triterpenoids also caused indirect inhibition of β-lactamases and reversed β-lactam resistance in MRSA (Catteau et al. 2017). To the best of our knowledge, ursolic acid was not reported to interact synergistically with colistin in bacteria. We are reporting for the first time that ursolic acid can reverse colistin MIC in eight non replicate clinical isolates of *Enterobacteriaceae* viz., *K. pneumoniae and E. coli.*

Colistin is a drug of last resort in *Enterobacteriaceae* (Falagas et al. 2005). In Gram negative bacteria, colistin is believed to cause outer membrane (OM) disruption by insertion of hydrophobic fatty acyl N terminal chain into outer membrane followed by entry into periplasm and thinning and lysis of inner membrane (Velkov et al. 2010). Presently colistin resistant strains are managed by combinatorial therapy involving various antimicrobial agents whose therapeutic efficiency is not well established (Cheng et al. 2018). A recent study has shown that use of outer membrane permeabilizing agents like anti protozoal drug pentamidine with Gram positive antimicrobial agents can indeed be highly effective against colistin resistant Gram negative bacteria (Stokes et al. 2017). Follow up study by the same group has shown that colistin, by virtue of its membrane permeabilizing effect, in combination with clarithromycin is highly effective against mcr-1 harboring *K. pneumoniae* both in vitro and in vivo (MacNair et al. 2018). Colistin doripenem combination was found to be quite effective in curtailing both colistin resistant and colistin heteroresistant *K. pneumoniae* isolates (Deris et al. 2012). In an effort to identify antibiotics that synergized with colistin against colistin resistant *Enterobacteriaceae*, among 20 different isolates tested, colistin in combination with linezolid, rifampin, azithromycin and fusidic acid exhibited synergistic activity against > 90% of the strains (Brennan-Krohn et al. 2018). Another recent study has shown that colistin sulphadiazine combination was synergistic and highly effective in curtailing 92.7% of 55 colistin resistant strains irrespective of mechanism of colistin resistance (Okdah et al. 2018).

In *A. baumannii* it was shown that colistins’ bactericidal effect was mediated by reactive oxygen species (ROS) especially ^•^OH radical and the agents that impair production of ROS were shown to delay killing effect of colistin (Sampson et al. 2012). In the present study since ursolic acid quenched ROS and the amount of ROS generated by colistin was further reduced in the presence of ursolic acid (Additional file 1: Fig S2), enhancement of colistins’ bactericidal effect in the presence of ursolic acid (Fig. 1 and Table 2) cannot be attributed to ROS. In the absence of ROS, the synergistic effect of colistin –ursolic acid combination could be primarily attributed to inhibition of colistin efflux by ursolic acid which is supported by real time efflux data (Fig. 4) and by the ability of ursolic acid to disrupt membrane potential in both *E. coli and K. pneumoniae* (Fig. 5). By perturbing membrane potential, which results in loss of proton motive force (PMF), efflux transport proteins that are powered by PMF are rendered non-functional. This is further supported by accumulation of dansyl tagged colistin within the cells (Fig. 6). In contrast, a previous study has shown that CCCP enhances bactericidal effect of colistin on *A. baumannii* cells but the authors contend that reduced metabolic activity due to CCCP, rather than efflux inhibition, is responsible for increase in bactericidal effect of colistin (Park and Ko 2015). In the present study, we have shown that intracellular concentration of dansyl tagged colistin increases only in presence of CCCP and ursolic acid (Fig. 6), implying that colistin accumulates in presence of either ursolic acid/CCCP. It is implied that increased intracellular colistin accumulation accounts for enhanced bactericidal effect observed in combination. Another recent study has shown that CCCP is effective in potentiating bactericidal effect of only colistin but not tigecycline and meropenem against multi drug resistant *Enterobacteriaceae* (Osei Sekyere and Amoako 2017). Although an earlier study reported that ursolic acid synergized with nalidixic adid by inhibiting acrAB pump in *E. coli* using in silico studies, the authors have not used acrA/acrB knock out mutants to prove that ursolic acid specifically inhibits acrAB pump in *E. coli* (Dwivedi et al. 2015). By virtue of its ability to disrupt proton motive force in both *E. coli* and *K. pneumoniae* as shown in the present study (Fig. 5), we can safely state that multiple efflux transporters that depend on PMF are inhibited concurrently, thereby preventing colistin efflux. Identifying pump involved in colistin efflux requires gene knock out mutants of all pumps present in both *E. coli* and *K. pneumoniae,* which is quite labor intensive. Alternatively, gene expression studies by qPCR can identify pumps that are upregulated only in colistin resistant bacteria, this can be followed by creating gene knock outs of only those pumps that are overexpressed, such an approach can help to identify pump(s) responsible for colistin efflux, which can be pursued as a future study. Compounds that act as protonophores like CCCP are typically toxic to higher eukaryotes (Itami et al. 2015; Park et al. 2018). By virtue of being a nutraceutical and based on our observations on brain and liver enzyme profiles (Fig. 2a, b) and histopathology studies (Fig. 2c), we can conclude that ursolic acid is non-toxic even at 2× concentration employed in present study, which gives credence for exploring it further as an adjuvant to colistin. A recent study has shown that curcumin can synergize with colistin and reduce persister formation in *A. baumannii* by increasing ROS, enhancing membrane permeability and by inhibiting efflux, which ultimately eliminates persisters (Kaur et al. 2018). In the present study too, we observed that ursolic acid exhibited synergy with colistin against *K. pneumoniae* and *E. coli* clinical isolates and its mode of action involved enhancing the outer membrane permeability and perturbing membrane potential that ultimately resulting in colistin accumulation, which enhances colistins’ attack on bacterial membranes. Although ursolic acid resembles curcumin in most of the other effects, by quenching ROS, it differs from curcumin and is probably slightly less effective. Hence future study would aim to make derivatives of ursolic acid that does not quench ROS, while retaining its ability to inhibit colistin efflux.

In conclusion from a library of 13 plant metabolites, we have screened and identified ursolic acid that interacts synergistically with colistin in clinical isolates of both *K. pneumoniae and E. coli*. By virtue of its synergistic interaction, ursolic acid caused colistin MIC reversal in both colistin resistant and colistin sensitive *Enterobacteriaceae* clinical isolates tested. Our observations revealed that ursolic acid was non-toxic and better potentiated bactericidal effect of colistin both in vitro, in a time kill assay and in vivo, in a zebrafish infection model against XDR strains U3790 and BC936 probably by enhancing access of colistin into the cells, which is achieved by perturbing outer membrane permeability and disrupting membrane potential that serves as a source of energy for multiple efflux pumps. Ability of ursolic acid to synergize with colistin highlights its therapeutic potential, which can be further enhanced by making derivatives that retain efflux inhibitory effect and gain ability of not quenching ROS. Ability of ursolic acid in potentiating bactericidal effect of last resort drugs like colistin is interesting and warrants further studies with improved derivatives in higher animal models.

## Additional file

**Additional file 1.** Additional tables and figures.

## Abbreviations

MTCC: microbial type culture collection
DMSO: dimethyl sulphoxide
MIC: minimum inhibitory concentration
FIC: fractional inhibitory concentration
UR: ursolic acid
*E. coli*: *Escherichia coli*
*K. pneumoniae*: *Klebsiella pneumoniae*
NPN: 1-*N*-phenylnaphthylamine
Disc3: 3,3′-dipropylthiadicarbocyanine iodide
ROS: reactive oxygen species
DCFH-DA: dichloro-dihydro-fluorescein diacetate
RTE: real time efflux
CCCP: carbonyl cyanide 3-chlorophenylhydrazone
PaβN: phenyl-arginine-beta-naphthylamide
AChE: acetylcholinesterase
XDR: extremely drug resistant
CFU: colony forming unit
OM: outer membrane
PMF: proton motive force
