# Supplementary material for: Ursolic acid inhibits colistin efflux and curtails colistin resistant Enterobacteriaceae
Source: AMB Express. 2019 Feb 18;9:27. doi: 10.1186/s13568-019-0750-4 (PMC6379497; doi:10.1186/s13568-019-0750-4)

AMB Express

# **Ursolic acid inhibits colistin efflux and curtails colistin resistant *Enterobacteriaceae***

Niranjana Sri Sundaramoorthy[1], Harihar M. Mohan[2], Shankar Subramaniam[3], Thiagarajan Raman[4], Subramaniapillai Selva Ganesan[3], Aravind Sivasubamanian[3] and Saisubramanian Nagarajan[1*]

[1]Center for Research on Infectious Diseases, SCBT, SASTRA deemed University, Thanjavur, Tamil Nadu, India

[2] Department of Microbiology and Immunology, University of Michigan Medical School, Ann Arbor, MI 48109, USA

[3]School of Chemical and Biotechnology, SASTRA deemed University, Thanjavur, Tamil Nadu, India

[4]Advanced Zoology and Biotechnology Department, Ramakrishna Mission Vivekananda College, Chennai, Tamil Nadu, Thanjavur

***Correspondence:**
Dr.SaisubramanianNagarajan
sai@scbt.sastra.edu

Niranjana Sri Sundaramoorthy – niran31sri@gmail.com

Harihar M. Mohan – hari9mm@gmail.com

Shankar Subramaniam – shanksu88@gmail.com

Thiagarajan Raman – thiagi200@yahoo.co.in

Subramaniapillai Selva Ganesan – selva@biotech.sastra.edu

Aravind Sivasubamanian - arvi@biotech.sastra.edu

**Table S1: Antimicrobial profiling *of Klebsiella pneumoniae* and *Escherichia coli* clinical isolates**

| Minimum Inhibitory Concentration (µg/ml) | | | | | | | | | | | |
|---|---|---|---|---|---|---|---|---|---|---|---|
| **Antibiotic/ Compound** | **MTCC KP** | **KP BC1994** | **KP BC2412** | **KP U3866** | **KP U2016** | **KP BC1415** | **KP U2192** | **KP E474** | **KP BC936** | ***E. coli* U3176** | ***E. coli* U3790** |
| **Ceftriaxone** | <1 | >128 | >128 | >128 | >128 | >128 | >128 | >128 | >128 | >128 | >128 |
| **Meropenem** | 0.5 | >32 | >32 | >32 | 32 | 16 | >128 | 16 | 16 | >32 | >32 |
| **Ciprofloxacin** | 4 | 32 | 16 | 128 | 16 | 16 | 32 | 32 | 16 | 64 | 256 |
| **Levofloxacin** | 1 | 8 | 16 | 64 | 32 | 32 | 64 | 32 | 32 | 32 | 32 |
| **Norfloxacin** | <2 | 64 | 16 | 128 | 64 | >128 | 64 | 128 | 64 | >128 | >128 |
| **Amikacin** | <2 | 4 | 16 | >128 | >128 | >128 | >128 | >128 | >128 | >128 | 8 |
| **Gentamicin** | <4 | 256 | <4 | >256 | >128 | >128 | >128 | >128 | >128 | >256 | 256 |
| **Tobramycin** | 16 | 64 | 64 | >256 | >128 | >128 | >128 | >128 | >128 | >256 | 128 |
| **Erythromycin** | 64 | 256 | 64 | 256 | >128 | >128 | >128 | >128 | >128 | >256 | 256 |
| **Tetracycline** | >128 | <2 | 4 | 16 | 8 | >128 | >128 | >128 | >128 | <2 | 128 |
| **Tigecycline** | >128 | >128 | >128 | >128 | >128 | >128 | >128 | >128 | >128 | >128 | >128 |
| **Colistin** | 2 | 2 | 1 | 1 | 8 | 8 | 8 | <2 | 32 | 1 | 32 |
| **Rifampicin** | 4 | 64 | 32 | >128 | >128 | >128 | >128 | >128 | >128 | 4 | 16 |
| **Streptomycin** | 64 | 128 | 32 | 4 | 32 | 32 | 32 | 32 | 32 | 32 | 16 |
| **Kanamycin** | >256 | >256 | >256 | >256 | >128 | >128 | >128 | >128 | >128 | >256 | >256 |

**Table S2: MIC of plant metabolites against XDR strains of *Klebsiella pneumoniae* and *Escherichia coli***

| | **Minimum Inhibitory Concentration (µg/ml)** | |
|---|---|---|
| **Compounds** | ***K. pneumoniae* BC936** | ***E. coli* U3790** |
| **Arjunolic Acid (AJ)** | 128 | 256 |
| **Acetyl Shikonin (AS)** | 512 | 256 |
| **B-Dimethyl acetyl shikonin (BD)** | 512 | 256 |
| **Caffeic Acid (CA)** | 128 | 512 |
| **Chrysin (CH)** | 512 | 1024 |
| **Crysophenol (CR)** | 512 | 512 |
| **Emoidin (EM)** | 512 | 512 |
| **Naringenin (NIN)** | 512 | 256 |
| **Naringin (NG)** | 64 | 256 |
| **Physicion (PH)** | 512 | 512 |
| **Ursolic acid (UR)** | 512 | 256 |
| **Ventilone A (VA)** | 512 | 256 |
| **Ventiloquinone (VQ)** | 512 | 512 |

**Fig S1: Membrane permeability of colistin resistant *Escherichia coli* and *Klebsiella pneumoniae* is enhanced by ursolic acid treatment.** Mid-log cells of *E.coli* U3790 and *K. pneumoniae* BC936 were treated with either colistin (Col) or ursolic acid (UR) or in combination (Col+UR) along with fluorescent dye N-Phenyl 1-Naphthylamine (NPN) and fluorescence intensity was measured using a spectrofluorimeter. NPN uptake factor was calculated as the ratio of background subtracted fluorescence of different groups to that of buffer.

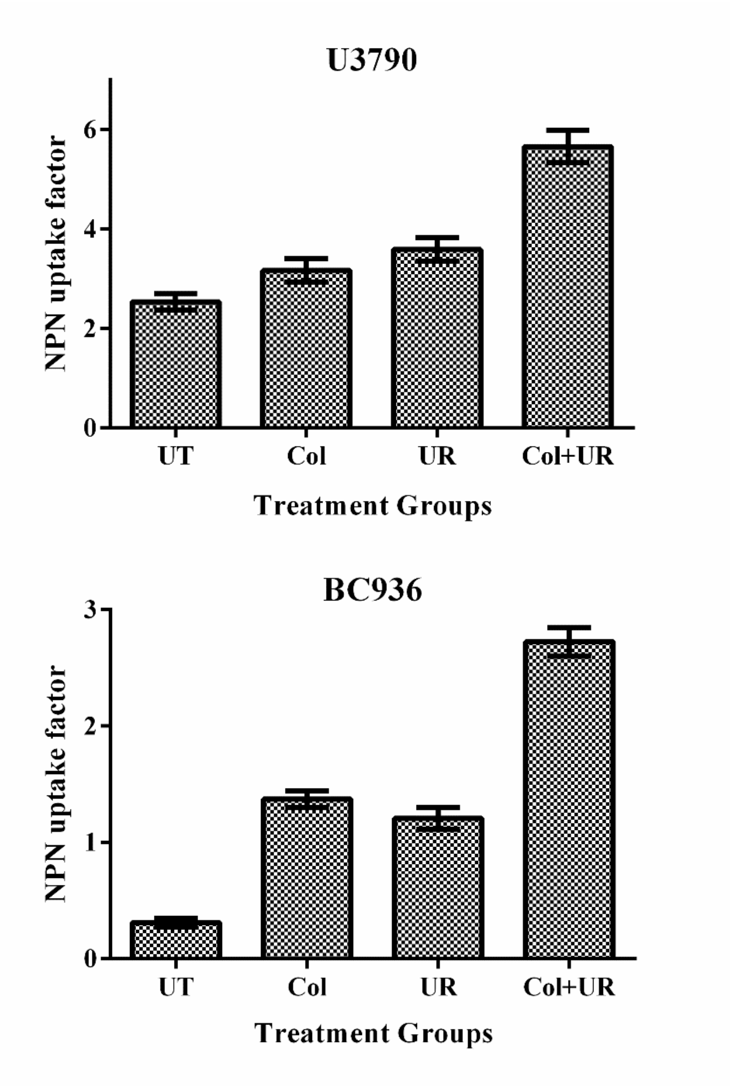

**Fig S2: Ursolic acid reduced ROS generation by colistin.** Mid-log cells of *E. coli* U3790 and *K. penumoniae* BC936 were challenged with treatments comprising colistin (Col), ursolic acid (UR) and colistin+ ursolic acid (Col+UR). $H_2O_2$ was used as a positive control in the assay. DCFH-DA was used as fluorophore to detect the amount of ROS generated in each of the treatments. The fluorescence intensity was quantified in a spectrofluorimeter.

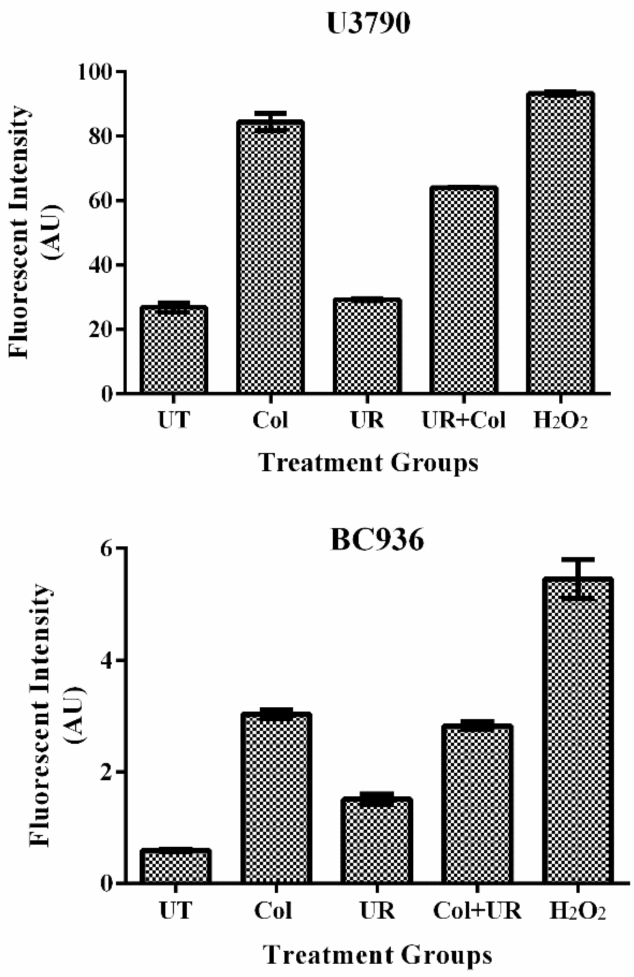

Supplement: Supplementary file 1 — Additional file 1. Additional tables and figures. [file 13568_2019_750_MOESM1_ESM.pdf]
